# An App-Delivered Self-Management Program for People With Low Back Pain: Protocol for the selfBACK Randomized Controlled Trial

**DOI:** 10.2196/14720

**Published:** 2019-12-03

**Authors:** Louise Fleng Sandal, Mette Jensen Stochkendahl, Malene Jagd Svendsen, Karen Wood, Cecilie K Øverås, Anne Lovise Nordstoga, Morten Villumsen, Charlotte Diana Nørregaard Rasmussen, Barbara Nicholl, Kay Cooper, Per Kjaer, Frances S Mair, Gisela Sjøgaard, Tom Ivar Lund Nilsen, Jan Hartvigsen, Kerstin Bach, Paul Jarle Mork, Karen Søgaard

**Affiliations:** 1 Department of Sport Science and Clinical Biomechanics University of Southern Denmark Odense Denmark; 2 Nordic Institute of Chiropractic and Clinical Biomechanics Odense Denmark; 3 National Research Centre for the Working Environment Copenhagen Denmark; 4 Institute of Health and Wellbeing University of Glasgow Glasgow United Kingdom; 5 Department of Public Health and Nursing Norwegian University of Science and Technology Trondheim Norway; 6 School of Health Sciences Robert Gordon University Aberdeen United Kingdom; 7 Department of Computer Science Norwegian University of Science and Technology Trondheim Norway

**Keywords:** low back pain, self-management, case-based reasoning, eHealth, mHealth, app, decision support system

## Abstract

**Background:**

Low back pain (LBP) is prevalent across all social classes, in all age groups, and across industrialized and developing countries. From a global perspective, LBP is considered the leading cause of disability and negatively impacts everyday life and well-being. Self-management is a recommended first-line treatment, and mobile apps are a promising platform to support self-management of conditions like LBP. In the selfBACK project, we have developed a digital decision support system made available for the user via an app intended to support tailored self-management of nonspecific LBP.

**Objective:**

The trial aims to evaluate the effectiveness of using the selfBACK app to support self-management in addition to usual care (intervention group) versus usual care only (control group) in people with nonspecific LBP.

**Methods:**

This is a single-blinded, randomized controlled trial (RCT) with two parallel arms. The selfBACK app provides tailored self-management plans consisting of advice on physical activity, physical exercises, and educational content. Tailoring of plans is achieved by using case-based reasoning (CBR) methodology, which is a branch of artificial intelligence. The core of the CBR methodology is to use data about the current case (participant) along with knowledge about previous and similar cases to tailor the self-management plan to the current case. This enables a person-centered intervention based on what has and has not been successful in previous cases. Participants in the RCT are people with LBP who consulted a health care professional in primary care within the preceding 8 weeks. Participants are randomized to using the selfBACK app in addition to usual care versus usual care only. We aim to include a total of 350 participants (175 participants in each arm). Outcomes are collected at baseline, 6 weeks, and 3, 6, and 9 months. The primary end point is difference in pain-related disability between the intervention group and the control group assessed by the Roland-Morris Disability Questionnaire at 3 months.

**Results:**

The trial opened for recruitment in February 2019. Data collection is expected to be complete by fall 2020, and the results for the primary outcome are expected to be published in fall 2020.

**Conclusions:**

This RCT will provide insights regarding the benefits of supporting tailored self-management of LBP through an app available at times convenient for the user. If successful, the intervention has the potential to become a model for the provision of tailored self-management support to people with nonspecific LBP and inform future interventions for other painful musculoskeletal conditions.

**Trial Registration:**

ClinicalTrial.gov NCT03798288; https://clinicaltrials.gov/ct2/show/NCT03798288

**International Registered Report Identifier (IRRID):**

DERR1-10.2196/14720

## Introduction

Low back pain (LBP) is a leading contributor to years lived with disability [1,2]. The economic costs associated with health care, sickness absence, lost ability to work, and treatment costs of nonspecific LBP are a major societal burden [3-5].

Clinical guidelines recommend education, exercise therapy, multidisciplinary treatments, and combined physical and psychological interventions for the management of LBP [6-10]. Self-management programs including elements of such recommended components are suggested as options for conditions like nonspecific LBP [11]. Self-management is commonly defined as active engagement and care for one’s own health by managing symptoms, physical and psychological problems, and their impact [11,12]. Although self-management is a recommended LBP treatment, the effectiveness of self-management for LBP has been reported in systematic reviews to be moderate for pain and small to moderate for pain-related disability [13,14]. These results may be explained by the large variation in the content of self-management programs [13] and the poor adherence commonly observed in relation to such programs [14,15]. Adherence is influenced by several factors such as tailoring of the program to the individual and support to persist with self-management [16].

Digital solutions such as mobile apps can be used as platforms for supporting self-management [17,18] and may solve some of the problems outlined above. First, some evidence indicates that tailoring of self-management advice to people with LBP may be more effective than nontailoring to improve pain and function [19]. Second, tailored digital health solutions may help to increase engagement and adherence [20]. During recent years, a vast number of apps that target self-management of LBP have been introduced to the commercial market. A systematic review identified 61 available apps on Google Play and the App Store and concluded that the apps were of poor quality and included poor-quality information from questionable sources and none of the apps had been tested for effectiveness [21]. A systematic review that synthesized and critically appraised the published evidence concerning the use of interactive digital interventions to support self-management of LBP found the literature to be heterogeneous and many studies to be poorly described [22]. Thus, the benefits and utility of digital interventions for self-management of LBP for the population at large remains unclear, presenting an important knowledge gap.

In the selfBACK project, we have developed an evidence-based and data-driven decision support system (DSS) delivered via a smartphone app to facilitate, improve, and reinforce self-management of nonspecific LBP. The design and implementation of the selfBACK DSS have been described elsewhere [23]. The selfBACK trial is designed as an international multicenter randomized controlled trial (RCT) with two parallel arms testing the effectiveness of the selfBACK DSS in addition to usual care (intervention group) versus usual care only (control group) for participants with nonspecific LBP. We hypothesize that participants randomized to the intervention group will have reduced pain-related disability at 3 months, measured by the Roland-Morris Disability Questionnaire (RMDQ), compared with participants randomized to the control group.

## Methods

### Participants and Setting

Inclusion and exclusion criteria are outlined in Textbox 1. The assessment of whether the criteria are considered to limit participation is performed either by the referring health care professional (HCP) or based on participant’s self-report. The selfBACK intervention is tested on a general LBP population rather than a specific subgroup to reflect that the intervention targets care-seeking patients not limited to specific characteristics such as symptom duration.

**Selection criteria.**

Inclusion criteria:

- Danish or Norwegian adults (aged 18 years and older)
- History of low back pain of any duration in patients having sought care for their low back pain within the preceding 8 weeks from primary practice (general practice, physiotherapy, or chiropractic serving as first point of contact) or a specialized outpatient hospital facility (Denmark)
- Must score mild to severe pain-related disability rated as 6 or above on the Roland-Morris Disability Questionnaire
- Must own and regularly use a smartphone with internet access
- Must have a working email address and access to a computer with internet access

Exclusion criteria:

- Unable to speak, read, or understand the national language (Danish or Norwegian)
- Cognitive impairments or learning disabilities limiting participation
- Mental or physical illnesses or conditions limiting participation as assessed by the referring health care professional or the participant
- Inability to take part in exercise or physical activity
- Fibromyalgia (diagnosed by a health care professional)
- Pregnancy
- Previous back surgery
- Ongoing participation in other research trials for low back pain management

### Recruitment and Screening

**Figure 1:**
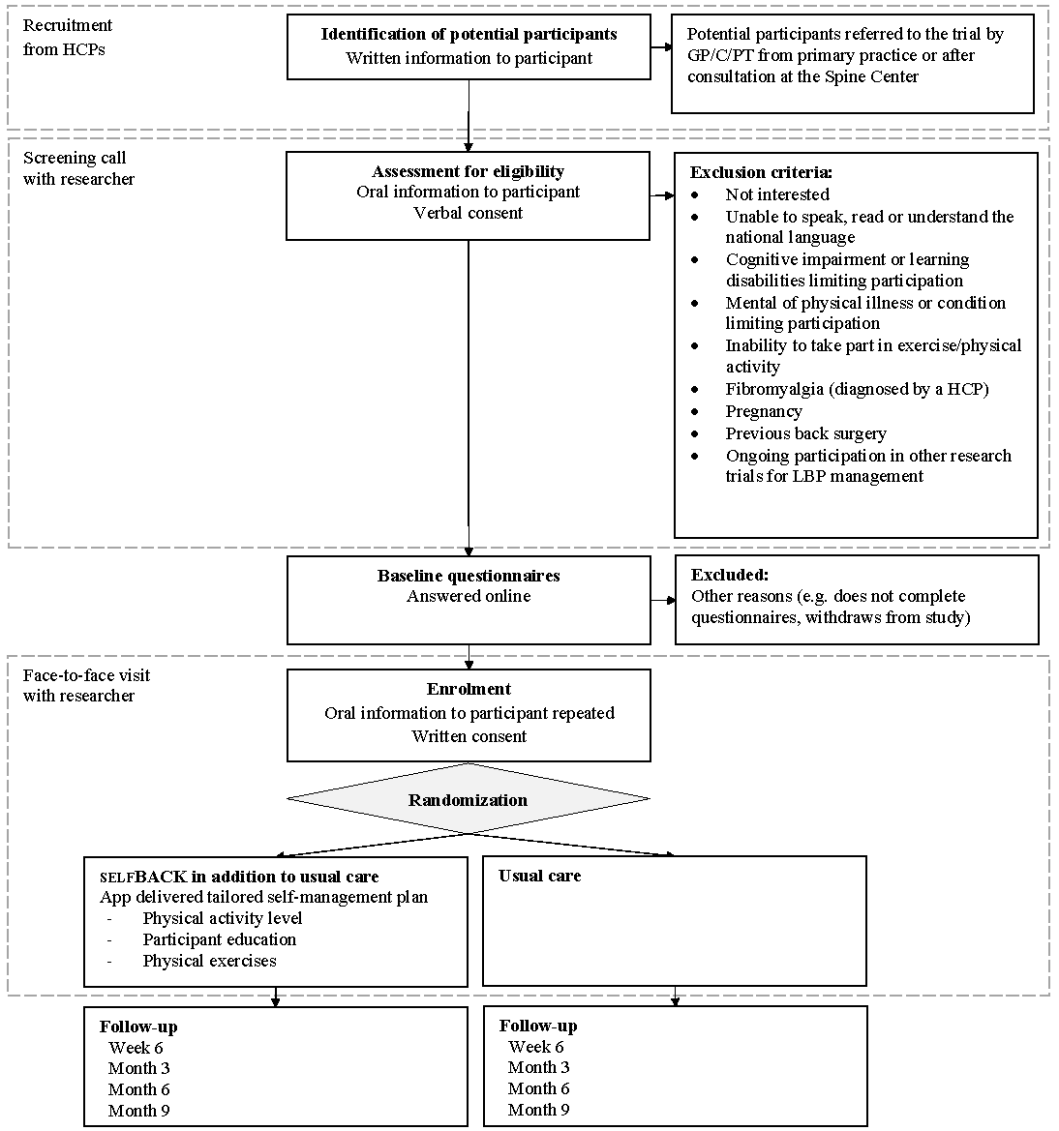
Participant flow through the selfBACK trial. The dashed lines indicate who the participant interacts with during the screening process and randomization. GP: general practitioner; PT: physiotherapist; C: chiropractor; HCP: health care professional; LBP: low back pain.

Recruitment is performed in Trondheim, Norway, and Odense, Denmark. The recruitment flow is described in Figure 1. A total of 350 participants are to be recruited to the RCT. Of these, 75% (262/350) will be recruited in Denmark and 25% (88/350) in Norway. Recruitment is undertaken by physiotherapists, chiropractors, and general practitioners. In Denmark, participants are additionally recruited from the Spine Centre of Southern Denmark, an outpatient hospital that provides care for people with back pain referred from primary care, either family physicians or chiropractors. The Spine Centre provides diagnostic assessment and prescribes treatment plans. For all recruitment sites, people seeking care due to nonspecific LBP may be referred to the trial by the consulting HCP based on a short description of eligibility for the trial. Final eligibility is assessed by the research team during a screening phone call. The recruitment to the selfBACK trial will not affect any planned routine diagnostic assessment or treatment (usual care).

Interested patients are screened via telephone by a member of the research team. If eligible and willing to participate, participants give their verbal consent to participate and are invited to complete the baseline questionnaire. Thereafter participants give their written consent to participate and are randomized to one of two groups.

### Randomization and Blinding

Participants are randomized to either (1) selfBACK DSS in addition to usual care or (2) usual care only. Randomization is performed as a block randomization with permuted blocks of random size and stratified by country and care provider (ie, general practitioner, physiotherapist, chiropractor, or Spine Centre). The allocation ratio between groups is 1:1. Randomization is performed in a Web-based trial management system (Web Case Report Form [WebCRF]) developed and administered by the Unit of Applied Clinical Research, Faculty of Medicine and Health Sciences, Norwegian University of Science and Technology (NTNU), Trondheim, Norway. The WebCRF system holds a minimal dataset on all screened participants (variables include trial indentification number, participant initials, country, type of HCP recruiting participant, age, and gender). The study is single-blinded; participants are not blinded to group allocation. Analysis and interpretation of the study results will be performed by researchers blinded to group allocation.

### Intervention

The trial and intervention are described following the Standard Protocol Items: Recommendations for Interventional Trials [24] and Consolidated Standards of Reporting Trials of Electronic and Mobile Health Applications and Online Telehealth (CONSORT-EHEALTH) [25] guidelines.

#### Usual Care

Participants receive usual care as deemed appropriate by their HCP. This includes any diagnostic procedure, treatment, or referral the HCP finds relevant considering the case history, clinical findings, and pragmatic, daily clinical practices. Participants can seek care, treatment, or help elsewhere as they find relevant. After the completion of the trial at 9 months, participants in this group are offered a wearable device like the one given to the selfBACK group.

#### Use of the selfBACK App in Addition to Usual Care

The selfBACK app presents participants with weekly tailored self-management plans consisting of recommendations on number of steps per day, educational material, and a program for strength and flexibility exercises. The process of tailoring the weekly self-management plan has been described elsewhere [23]. In short, a weekly self-management plan is created based on information from four different sources: (1) the baseline questionnaire; (2) a weekly question and answer session (tailoring session) where the participant via the app provides up-to-date information on their LBP, function, fear-avoidance, sleep, pain self-efficacy, perceived stress, symptoms of depression, and barriers for self-management; (3) the participant’s report on accomplishing the recommended program for strength and flexibility exercises in the preceding week; and (4) number of steps in the preceding week recorded by a physical activity–detecting wristband connected to the selfBACK app. Tailoring of the self-management plans is achieved by using case-based reasoning (CBR) methodology. CBR is a branch of artificial intelligence that imitates human reasoning and tries to solve new problems by reusing solutions that were applied to past similar problems. Hence, in the selfBACK DSS, the CBR system uses data about the current participant case (from the sources described above) along with knowledge about previous and similar participant cases to tailor the self-management plan to the current individual with LBP. The intervention is not intended to replace follow-up by an HCP but to supplement the usual care, and the participant is informed accordingly. Using the CBR methodology to support self-management is relatively unexplored. A recent study showed that using the CBR methodology has the potential to improve glycemic control in type 1 diabetes [26,27]. However, we are not aware of any studies that have used CBR to support self-management of musculoskeletal disorders.

The content for the app was developed using an intervention mapping process [28]. Full details of the process will be reported separately. During the intervention mapping, the content of the app was reviewed and assessed by patients and clinicians and the app was then tested in two separate feasibility and one pilot study before the RCT version of the app was finalized. The results from these studies will be reported separately. Participant experiences using the app and entering the studies were captured in interviews and informed the conduct of this RCT. Overall, the app was very well received among the pilot users, and feedback from participants gave us areas for improvement for the RCT (eg, explanation text in the app and during installations). The self-management plans are built from three types of content: (1) a bank of educational material, (2) a bank of strength and flexibility exercises, and (3) physical activity level (ie, step count). An overview of the available content is presented in Table 1. The educational material is structured under 14 main categories. Short messages are about 140 characters long. Some messages may include links to longer, more explanatory texts (maximum 500 characters) or tools that can be used to help the self-management of LBP. Some short messages are rewritten into quizzes, where the educational content is rephrased into yes or no questions.

The bank of physical exercises holds 56 strength and flexibility exercises organized in 5 targets and 14 pain-relief exercises (Table 1). Exercises are presented as a short video accompanied by a written instruction. The default recommendation is to perform exercises in 3 to 5 sessions per week of 15 minutes duration (eg, 3 exercises with an estimated duration of 5 minutes per exercise, Table 1). The number of exercises is adjusted by the participant’s indication of time available. The participant reports on completed number of sets and repetitions per exercise. The progression and regression of exercise difficulty is based on the reported completion level. If the participant reports a flare-up of LBP in the weekly tailoring session, a set of pain-relief exercises is recommended instead of strength and flexibility exercises.

Physical activity is tracked using a wearable device (Mi Band 3, Xiaomi). The wristband shows the achieved step count per day. Educational messages and notifications aimed to motivate more physical activity are pushed to the participant through the app based on the step count data.

**Table 1:** Overview of the content of self-management plans.

Data available	Physical activity	Physical exercise	Education
Information from preceding week	Achievement of preceding week’s step goal	Completion of exercise sessions	Completion of educational messages and quizzes
Content available	Physical activity registration: Step count registration by wristband Individualized feedback for daily, weekly, and monthly step count Advice to stay active Motivational messages to increase physical activity	Exercise targets: Abdominals Back extensors Core stability Gluteal and hip muscles Flexibility Pain relief Default program: Three exercises (1 abdominal + 1 back extensor OR 1 core stability exercise). Remaining exercises chosen randomly from the other groups.	Message themes: Information about LBP^a^ Understanding mind-body connection Self-management for LBP Thoughts, behavior, attitude, and feelings Fitting in self-management in a busy life First aid when your back hurts LBP and comorbidities Goal-setting and action planning Pacing and progression Problem-solving Relaxation Sleep and LBP Social support Overcoming barriers for self-management Educational tools: Sleep reminder and sleep hygiene Mindfulness Goal-setting

^a^LBP: low back pain.

### Outcomes

The primary outcome is pain-related disability at 3-month follow-up assessed using the RMDQ [29]. The questionnaire includes 24 items asking participants to indicate if they experience functional impairments by answering yes or no to a series of descriptions of functional abilities. Higher scores indicate higher level of disability [30]. For the selfBACK trial, we aim to identify a 2-point difference in RMDQ between the intervention and control group at 3-month follow-up. The rationale for selecting this cutoff was based on several considerations. First, self-management through selfBACK is included as an add-on to usual care in this trial. Although the magnitude of effect for this novel intervention is difficult to predict, a small beneficial effect above that of usual care could be important for this group of patients. Second, the suggested minimal clinically important difference in RMDQ may vary according to the disability level in the population under study [31]. Even though a 5-point difference has been reported as clinically important [32], others have suggested a 1- to 2-point difference to be clinically important if the disability level is low [33].

Descriptive variables include age, gender, height, weight, and report of any comorbidities (comorbidities were registered using an existing questionnaire (HUNT3) from the Norwegian HUNT study [34]). Demographic variables including family relations, ethnicity, educational status, employment, and work characteristics if employed are collected at baseline (Table 2).

A range of secondary and exploratory outcomes is included in the trial, and participants randomized to use the selfBACK app in addition to usual care are asked a set of tailoring questions weekly to individualize their self-management plan (Table 2). App use data such as number of visits, duration spent using the app, achievement scores, and number of days with visits are registered (Matomo software).

**Table 2:** Overview of the information collected at baseline, during the weekly tailoring sessions, and at follow-ups at 6 weeks and 3, 6, and 9 months.

Characteristics		Baseline	Weekly tailoring	Follow-ups
**Descriptive variables**				
	Participant characteristics	x		
	Sociodemographics	x		
**Primary outcome**				
	Roland-Morris Disability Questionnaire [29,32]	x		x
**Secondary and exploratory outcomes**				
	Average pain intensity past week	x	x	x
	Worst pain intensity past week	x		x
	Duration of current episode with low back pain	x		x
	Pain medication frequency past week	x		x
	Fear-Avoidance Belief Questionnaire [35]	x	x^a^	x
	Pain Self-Efficacy Questionnaire [36]	x	x^b^	x
	Activity limitation, work and leisure	x		x
	Work ability index (single-item) [37]	x	x	x
	Saltin-Grimby Physical Activity Level [38]	x		x
	Patient Specific Function Scale [39]	x		x
	Sleep problems [40]	x	x^b^	x
	Perceived Stress Scale [41]	x	x^b^	x
	Quality of life: EuroQoL 5-Dimension [42]	x		x
	Brief Illness Perception Questionnaire [43]	x		x
	Patient Health Questionnaire–8 [44]	x	x^b^	x
	Global Perceived Effect			x
	Patient Acceptable Symptom State			x
	Perceived barriers		x	
	Pain-related function		x^c^	

^a^Fear-avoidance assessed with single-item Tampa scale [45].

^b^Reduced number of items or single items.

^c^Function assessed with single items from Chronic Pain Grade Scale [46].

### Data Collection, Storage, and Protection

Outcome measures are collected at baseline, 6 weeks and 3, 6, and 9 months. Data collection is Web-based, and all data are entered directly into the selfBACK database by the participants. To maximize response rate, reminder emails are sent after 3 days and again after 6 days if no response to the first email. If still no answer, a researcher will contact the participant and ask if they are willing to answer the RMDQ questionnaire over the phone at follow-ups.

All outcome and other data are stored on secure servers at NTNU, the servers are firewall protected, and back-up is performed daily. Data storage is consistent with national (Denmark and Norway) and European regulations on data protection. Also, all data transferring processes are protected using https and Secure Sockets Layer as well as sending the data in encrypted format.

### Sample Size

The sample size calculations have been performed in two ways. First, we performed a calculation assuming only one follow-up measure and a standard deviation of the RMDQ score of 6 points. The expected standard deviation was informed by previous high-quality studies in Denmark and United Kingdom investigating similar LBP populations [47-50]. Based on this calculation, we estimated that a sample size of 382 (191 in each group) was necessary to detect a 2-point difference with 90% power and a 2-sided alpha level of .05.

Second, we performed a simulation using 1000 repetitions of a mixed-model regression for repeated measures assuming (1) 3 data points per participant (ie, baseline, 6 weeks, and 3 months), (2) a 2-point difference between groups on RMDQ at 3 months, (3) a standard deviation of 6 points, and (4) a correlation between repeated measures of 0.4. The latter was based on information from previous trials with repeated measures for the RMDQ in similar LBP populations [51,52]. Based on these assumptions and an alpha level of .05, sample size calculations show that 250 participants (ie, 125 participants in each group) result in a power of 92% (95% CI 90%-93%) to detect a 2-point difference in RMDQ between the intervention group and control group at 3 months. Furthermore, simulations assuming a 2-point difference between groups observed at both follow-up time points (6 weeks and 3 months) indicated that a sample size of 180 (90 in each group) will result in a power of 94% (95% CI 92%-95%). These sample size calculations indicate that a sample size of approximately 250 persons (125 in each group) is adequate when using the repeated measure design. A recent systematic review showed that attrition rates ranged between 4% to 94% for digital self-management interventions lasting between 2 weeks and 12 months in LBP populations [22]. To allow for a 30% dropout rate at 3-month follow-up, we aim to include a total of 350 participants in the trial (175 participants in each arm).

### Statistics

The primary analysis will estimate mean group difference with 95% confidence interval of the RMDQ score over the first 3 months. Analyses will be conducted according to the intention-to-treat principle using a linear mixed model for repeated measures. This model includes all available data for all participants at each time point (ie, baseline, 6 weeks, and 3 months). In the regression model, individual participants will be specified as a random effect, accounting for the within-subject covariance structure. The effect of group and time will be specified as fixed effects using a joint variable of intervention and time. The analysis will investigate the effect of the intervention as constant over time, as well as an interaction between time and group allocation. Here, baseline levels are pooled over the two study groups assuming that any baseline differences are due to chance [53]. All effects will be estimated both crude and adjusted for the two variables used for stratification in the randomization (ie, country and care provider) [54]. Any missing values are inherently accounted for in the mixed-model approach [55].

To increase transparency, a statistical analysis plan will be agreed upon and made publicly available before ending the collection of the primary outcome. To reduce the risk of biased interpretation of results, the following procedure will be undertaken: two interpretations will be drafted based on a review of the primary outcome data with groups arbitrarily labeled A and B [56]. One interpretation assumes that A is the intervention group and B the control group, the other interpretation assumes the reverse. After agreeing on both interpretations, the randomization code is broken and the correct interpretation chosen.

### Process Evaluation

A process evaluation exploring how participants use the intervention in daily life will be conducted as an integrated part of the RCT. For this we will use a mixed-methods process evaluation: gathering quantitative measures by questionnaires for participants including the Virtual Care Climate Questionnaire [57] and 3 rating questions (overall rating of the app, ease of use, recommendable to others), measures of data analytics on app use, and semistructured qualitative interviews. Normalization process theory [58], an implementation theory used extensively to identify barriers and facilitators to uptake and use of new technologies [59], will provide the conceptual underpinning to the process evaluation. The process evaluation will be guided by the RE-AIM framework and investigate all 5 elements of the framework: reach, effectiveness, adoption, implementation, and maintenance [60]. The full details on design and methods for the process evaluation will be published separately.

### Ethics and Dissemination

The trial was approved by the national ethical committees in Denmark (S-20182000-24) and Norway (2017/923-6). Correspondingly, national review boards or data protection agencies have approved the trial. In Denmark, approval was granted from the Danish Data Protection Agency through application to the University of Southern Denmark’s legal office (201-57-0008) and in Norway from the National Data Protection Authority or the Centre for Research Data through the ethics approval. The trial is registered with ClinicalTrials.gov [NCT03798288].

The trial results will be reported in accordance with the CONSORT 2010 reporting guideline and the 2013 CONSORT-EHEALTH checklist amendment for reporting Web-based and mobile-based RCTs [25,61].

No serious adverse events are expected for this trial. Should a participant contact the research team concerning any worsening of symptoms, the participant will be advised to seek care from their HCP as they normally would. All inquiries regarding potential adverse events will be recorded and discussed in an internal audit and reported with the study results. In addition, the selfBACK DSS is designed to react to increased pain or deterioration in symptoms, and it will adjust the self-management plans based on this information. In addition, participants are informed in the written information and during the screening call and inclusion process that this intervention is an add-on to usual care and should not replace contact with their HCP and that they should always follow the advice of the consulting HCP. Also, the app contains a Caution section describing worsening in symptoms that should be acted upon and advising participants to seek care from their consulting HCP if they experience any such symptoms.

## Results

Recruitment to the trial started in early 2019 and is expected to run until the end of 2019. Data collection is expected to be complete by September 2020, and dissemination of trial results is planned thereafter. The results on the primary outcome is expected to be ready during fall 2020.

## Discussion

This protocol describes the design and methods of the selfBACK trial assessing the effectiveness of the selfBACK app in addition to usual care in helping people with nonspecific LBP manage their condition. Digital solutions have been described as promising platforms for supporting people in managing chronic conditions [17,18], and a vast number of mobile apps for managing LBP are already available on the commercial market [21]. In a recent systematic review, 9 studies were identified describing digital mHealth and eHealth self-management interventions for the LBP population [22]. Few studies reported their theoretical underpinnings for the included content, and consequently, the evidence base for digital self-management interventions for LBP remains weak [22,62]. Two recent RCTs showed improvements in participants’ symptom status after 12 weeks of using apps providing a digital program of noninvasive treatment options for LBP [63,64]. Only the study by Shebib and colleagues [63] reported greater improvements for the intervention group than the control group. However, the choice of comparators in the two trials were markedly different. In the study by Shebib and colleagues [63], the control group was given a static program consisting of 3 digital educational articles whereas participants in the intervention arm had unlimited access to a personal coach. In the other RCT, no personal contact was present in the intervention arm, but the comparator was individual lessons with a physiotherapist.

The content of the selfBACK intervention was developed using an intervention mapping process and is therefore theoretically underpinned and evidence-based [23]. Also, the DSS is a data-driven system that uses CBR methodology to structure and reuse real participant information to give advice and guide the self-management process in new participant cases. Thus, over time the DSS learns from experience which results in improved self-management plans for future participant cases. In addition to the learning from participant cases, a set of carefully described rules was developed to tailor the self-management plans to different scenarios (eg, flare-up of LBP). We also used participant cases derived from existing patient cohorts to develop a set of seed cases for the case base. Additionally, the app was tested in a pilot study before the start of the RCT, and these participants cases were included in the case base. This ensures clinically meaningful cases in the case base at the start of the RCT.

It is important to recognize that the content of usual care will differ for participants both within and across study centers (countries) of this trial. This is a common problem in trials where usual care is the comparator. However, it is also a reflection of how LBP is managed in a real-life setting. Thus, the results of the trial will have a high degree of external validity. In addition, the process evaluation for the trial will address perceptions of usual care through interviews with participants from the usual care group as well as with participants using the selfBACK app.

Similarly, the content of the suggested self-management plans will vary for participants using the selfBACK app. The app presents tailored self-management plans with three components: exercise, physical activity, and educational material. However, it is very likely that some components will appeal more to some participants than others. Therefore, should the RCT show the selfBACK app in addition to usual care to be more effective than usual care only, the trial design does not allow analyses of which components of the intervention may be causal of such an effect, although the process evaluation may provide some useful insights regarding such issues.

The outcomes from this trial will provide valuable new insights into the potential of mHealth solutions to support effective self-management in relation to LBP, while the parallel process evaluation will aid understanding of barriers and facilitators to uptake, use, and wider implementation of the intervention. The effectiveness of the app will be evaluated on the primary outcome; however, a range of secondary outcomes is included to elucidate the variation in and complexity of symptoms in people with LBP.

## Abbreviations

CBR: case-based reasoning
CONSORT-EHEALTH: Consolidated Standards of Reporting Trials of Electronic and Mobile Health Applications and Online Telehealth
DSS: decision support system
GLA: University of Glasgow
HCP: health care professional
LBP: low back pain
NFA: National Research Centre for the Working Environment
NTNU: Norwegian University of Science and Technology
RCT: randomized controlled trial
RGU: Robert Gordon University
RMDQ: Roland-Morris Disability Questionnaire
UoSD: University of Southern Denmark
WebCRF: Web Case Report Form
